# Detection of the Leprosy Agent *Mycobacterium lepromatosis* in South America and Europe

**DOI:** 10.4269/ajtmh.16-0713

**Published:** 2017-01-11

**Authors:** Xiang Y. Han

**Affiliations:** 1 Clinical Microbiology Laboratory, The University of Texas, MD Anderson Cancer Center, Houston, Texas. E-mail: xhan@mdanderson.org

Dear Sir:

I read with interest the recent article “Two Cases of Leprosy in Siblings Caused by *Mycobacterium lepromatosis* and Review of the Literature” by Sotiriou and others.^1^ I noted omission of a 2014 article that reported detection of *M. lepromatosis* in Brazil and Myanmar.^2^ The omission made the literature review incomplete, and more relevantly, failed to recognize wider geographic distribution of the agent that includes South America and Asia in addition to North and Central America. Worldwide, Brazil has the highest incidence of leprosy (1.7 per 10,000 population) with 33,955 new cases recorded in 2011.^3^

The 2014 article provided a systematic analysis of 96 leprosy cases from four countries.^2^ We confirmed an etiologic agent in 46 of the 52 patients from southern Brazil. Of the 46 patients, *Mycobacterium leprae* was detected in 36, *M. lepromatosis* in seven, and both agents in three. Thus, *M. lepromatosis* caused or contributed to 21.7% of cases. Methodologically, the species-specific polymerase chain reaction amplicons were all single band of designed length, and amplicons from two representative cases were sequenced to further verify specificity. The sequence of one amplicon was deposited in GenBank (http://www.ncbi.nlm.nih.gov/nuccore/GQ900374). We also detected *M. lepromatosis* in two of six patients from Myanmar, in accord with prior detection in Singapore.^4^

The editorial by Scollard^5^ accompanying the Sotiriou and others article was informative. However, the suggestion to lump *M. lepromatosis* and *M. leprae* as “*M. leprae* complex” is at odds with a genome-scale mismatch of ∼13% that was revealed recently.^6^^,^^7^ Intuitively, given that both organisms cause leprosy and are uncultivable so far, one tends to bundle them. But the genetic gap is simply too big to overlook. Somewhat analogously, the genomes of human and chimpanzee differ by ∼3%, and we cannot call us “human complex” or “chimpanzee complex.” By contrast, the term “*Mycobacterium tuberculosis* complex,” used in diagnostic microbiology, is appropriate because those organisms not only exhibit similar pathogenicity and phenotypic features but also have identical 16S rRNA genes and differ minimally (∼0.05%) in genome sequences. Microbiology today is defined by the inseparable features of morphology, culture characteristics, and genetics of a microbe.

The report of leprosy in red squirrels in Scotland is intriguing to note, particularly the 99% genetic match of its agent with *M. lepromatosis*.^8^ The study suggests likely presence of *M. lepromatosis* in Europe in addition to the Americas and Asia, and raises the question of why there have been no reports of human infections with *M. lepromatosis* in Europe.

Similar mycobacterial dermatitides in cows in France and in cats in Australia have been reported.^9^^,^^10^ The study of the cow agent analyzed portions of six conserved genes totaling 3,231 nucleotides.^9^ Judged from the GenBank deposits (KJ095004–KJ095009), the five protein-coding genes matched 88–93% in sequence with those of *M. leprae* and/or *M. lepromatosis*, and the *16S* gene matched best with *M. lepromatosis* (98.4% identity). The cat agent was analyzed with a 556-bp segment of the *16S* gene (AJ294740–AJ294746),^10^ with best matches with *M. leprae*, *Mycobacterium haemophilum*, and *Mycobacterium malmoense* (all 96.4–96.6%). Therefore, the cow and cat agents are likely two new *Mycobacterium* species. Thus far, none of the animal agents has been cultivated. Whether they contain pseudogenes—the hallmark of the leprosy bacilli—remains to be seen.
